# COVID-19 experiences of social isolation and loneliness among older adults in Africa: a scoping review

**DOI:** 10.3389/fpubh.2023.1158716

**Published:** 2023-05-09

**Authors:** Isaac Akinkunmi Adedeji, Andrew Wister, John Pickering

**Affiliations:** ^1^Department of Gerontology, Simon Fraser University, Burnaby, BC, Canada; ^2^Gerontology Research Centre, Simon Fraser University, Burnaby, BC, Canada

**Keywords:** Africa, COVID-19, loneliness, older adults, social isolation

## Abstract

**Objective:**

Social isolation and loneliness (SI/L) are considered critical public health issues. The primary objective of this scoping review is to document the experience of SI/L among older adults in Africa during the COVID-19 pandemic, given research gaps in this area. We identified the reasons for SI/L, the effects of SI/L, SI/L coping strategies, and research and policy gaps in SI/L experiences among older adults in Africa during COVID-19.

**Methods:**

Six databases (PubMed, Scopus, CINAHL, APA PsycINFO, Web of Science, and Ageline) were used to identify studies reporting the experiences of SI/L among older adults in Africa during the COVID-19 lockdown. We adopted the Joanna Briggs Institute (JBI) methodology and the Preferred Reporting Items for Systematic Reviews and Meta-Analyses extension for Scoping Reviews (PRISMA-ScR).

**Results:**

Social isolation and loneliness due to COVID-19 in Africa affected older adults' mental, communal, spiritual, financial, and physical health. The use of technology was vital, as was the role of social networks within the family, community, religious groups, and government. Methodological challenges include the risk of selective survival bias, sampling biases, and limited inductive value due to context. Also, lack of large-scale mixed methods longitudinal studies to capture the experiences of older adults during COVID-19. There were essential policy gaps for African mental health support services, media programs, and community care service integration targeting older adults in the era of the COVID-19 lockdown.

**Discussion:**

Like in other countries, COVID-19 lockdown policies and the lockdown restrictions primarily caused the experience of SI/L among older adults in Africa. In African countries, they resulted in a severance of older adults from the cultural structure of care for older adults and their familial support systems. Weak government intervention, personal situations, challenges regarding technology, and detachment from daily activities, disproportionately affected older adults in Africa.

## Introduction

Globally, COVID-19 accounted for a disproportionate number of deaths among older adults, and in Africa, 50% of COVID-19 deaths occurred among older adults aged 60 and over (1). Consequently, the outbreak of COVID-19 and the restrictions imposed to contain the spread of the disease created unanticipated disruption to social life, leading to myriad physical, social, and mental health challenges. These disruptions significantly affected older adults, since the disease risk was higher, resulting in more stringent behavioral adjustments. In addition, since social and physical distancing was not commonplace in Africa's more communitarian social environment (i.e., more multigenerational households), the requirements for COVID-19 control were sudden and different than in many other countries (2). Additionally, the conditions impacted life differently, especially according to urban, rural milieux, and kinship structures. Older adults in Africa may also have been more vulnerable to social isolation and loneliness (SI/L) due to their family-based approach to care and living arrangements [e.g., (3, 4)].

Research indicates that SI/L are related but distinct concepts. SI refers to reductions in the quantity and quality of social interaction and network of support. In contrast, loneliness is a mental state that occurs due to the lack of quality social connections necessary to meet the social needs of a person (5). Ideally, to stay socially connected and receive adequate support, older adults need to be embedded in supportive social networks. However, in Africa, geriatric care relies heavily on kinship systems (3, 6). At the same time, formal service approaches tend to be weak or non-existent because of a lack of funding and political will (7).

SI/L are considered critical public health issues often associated with poor physical and mental health, lower psychological wellbeing, and mortality (8–12). Specifically, SI/L accounts for a significant risk of depression (50%) and anxiety (47%) across studies in different countries (13). In addition, SI/L are associated with an increased risk of physical health problems such as frailty and chronic conditions (14, 15), and about 30% risk of heart disease and stroke (16). In Africa, these associations have not been closely examined due to paucity of quantitative research on the experiences of older adults.

However, SI/L became a focal public health challenge during the pandemic in many countries, with the rate of loneliness ranging from 20 to 40% in China, Europe, Latin America, Canada, the US, and Africa (17–19). Moreover, the rate of SI/L varies across social settings, and it is higher in social contexts where people feel socially disconnected—such as in residential care (20). Thus, in the African context, the cultural approach to care, a sense of community, kinship and living arrangements, and the African notion of shared ties, are significant factors for lower levels of SI/L in these community contexts (21). Unfortunately, these ties were tested during the pandemic because the cultural approach to care requires face-to-face interactions. Thus, the inability of older adults to receive adequate physical, emotional, and material support from families and friends during the pandemic was a significant cause of strain on their social and mental health (22). During the pandemic, in many countries with more developed infrastructures, the use of technology, such as phone and other low-tech communicative devices, have been shown to be beneficial in reducing SI/L and supporting some older adults to stay socially connected (23, 24). The uptake of information and communication technology appeared to increase among older adults in some countries, but particularly in places where there was technology uptake prior to the pandemic (24, 25). However, the extent to which telephone reduced SI/L among older adults in Africa is unclear because few studies have explored this area. In addition, there is a “gray” digital divide between rural and urban settings, and materially deprived environments, making research contexts unique (14, 22). For instance, digital infrastructure such as connectivity, cost of data and devices, and access to energy supply in most parts of Africa contribute to a digital divide (26). Furthermore, the weakness of resources that the government provides, including pensions, palliative care, health care, and community supports, may have weakened system-level resilience (27) in Africa. Yet, there appeared to be some successful approaches, such as religious groups that fostered social connection and support by organizing small groups to meet on a face-to-face rather than large congregation basis and to provide food to older adults who were lacking (28, 29).

Overall, among the 54 African countries, there is limited empirical evidence that comprehensively explores the experience of SI/L among older adults, in particular, during the pandemic. Therefore, the primary objective of this scoping review is to document the experience of SI/L among older adults in Africa during the COVID-19 pandemic. The secondary objectives are to: (1) identify the reasons for SI/L among older adults in Africa due to COVID-19 restrictions, (2) highlight the effects of SI/L among older adults during COVID-19 restrictions, (3) describe the SI/L coping strategies, including social support adopted during the COVID-19 restrictions, (4) discuss the research and policy gaps of SI/L experiences among older adults in Africa in the era of COVID-19.

## Methods

We conducted a scoping review of studies reporting the experiences of SI/L among older adults in Africa during the COVID-19 lockdown. We adopt the Joanna Briggs Institute (JBI) (30) methodology and the Preferred Reporting Items for Systematic Reviews and Meta-Analyses extension for Scoping Reviews (PRISMA-ScR) (31).

### Search strategy

We searched six databases for research articles exploring how older adults coped with SI/L during the COVID-19 restrictions in Africa. The focus was on empirical research articles because they provide evidence from older adults' experiences of SI/L during the pandemic. We searched PubMed (2020—to week 4 of May 2022); Scopus (2020—week 4 of May 2022); CINAHL Complete (2020—week 4 of June 2022); APA PsycINFO (2020—week 2 of July 2022); Web of Science (2020—week 4 of July 2022); Ageline (2020—week 4 of July 2022). We used operators and took cognizance of MeSH (Medical subject heading) terms and their associated approaches. There were variations across the broad terms—“Africa” AND older AND adults OR aged OR elderly OR seniors AND social AND isolation OR loneliness AND covid-19 OR coronavirus OR 2019-ncov OR SARS-CoV-2 OR cov-19 (see Appendix 1).

### Inclusion and exclusion criteria

Studies were selected based on the following inclusion criteria: (1) The article focuses on Africa, (2) Written in the English Language, (3) It specifically focuses on exploring the experiences of SI/L among older adults, (4) Was conducted in Africa, among African older adults who are 50+, and we considered this lower age cap due to the life expectancy at birth of African older adults. We also considered the fact that the higher age cap (60+) leads to the exclusion of older adults from surveys (1), (5) Is based on empirical data, and (6) Was focused on the COVID-19 pandemic. Studies were excluded if they did not fit these inclusion criteria.

**Figure 1:**
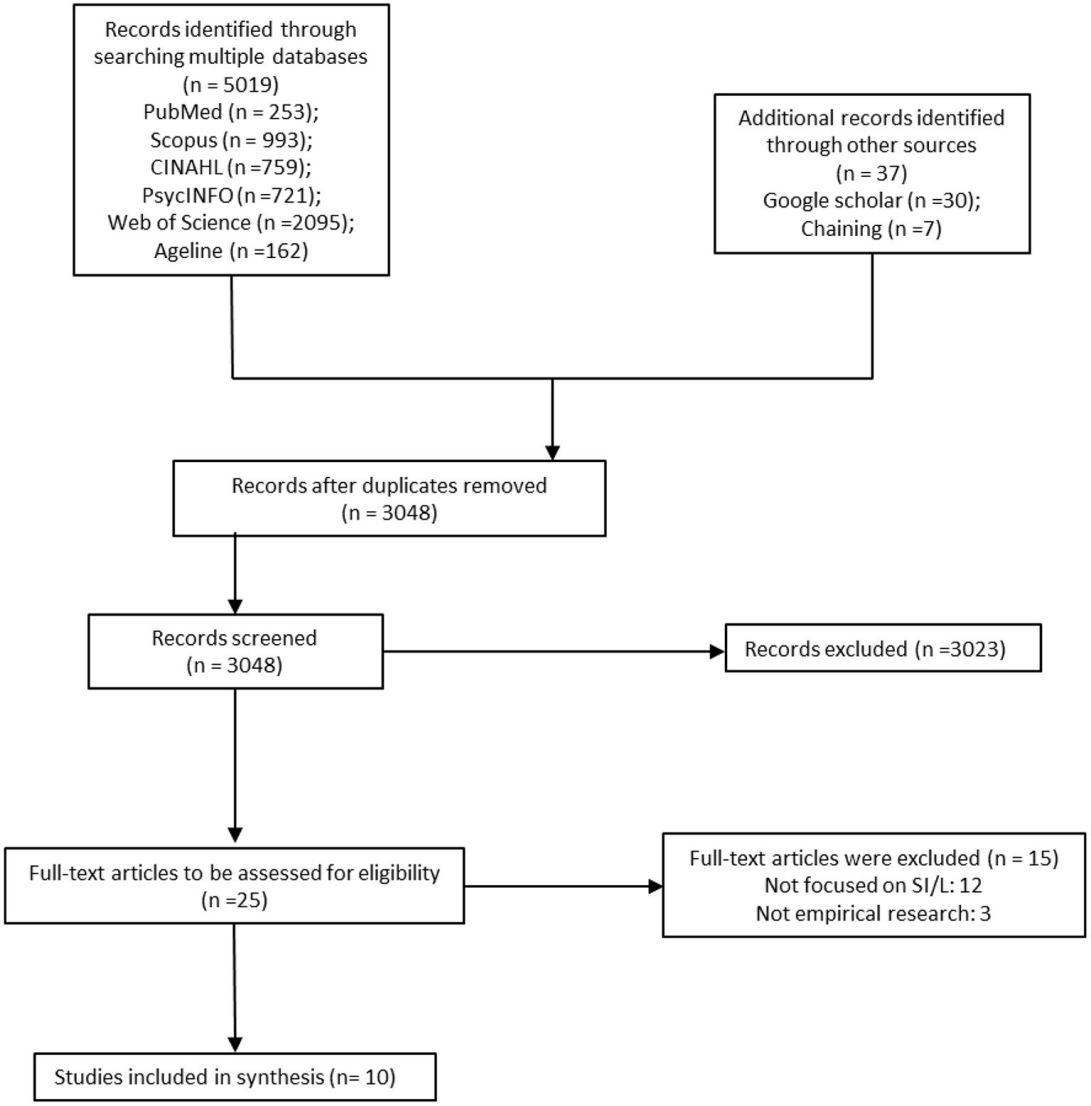
PRISMA flow diagram illustrating the search strategy.

We initially retrieved a total of 5019 articles from PubMed (253), Scopus (993), CINAHL (795), APA PsycINFO (721), Web of Science (2095), and Ageline (162). After de-duplication, there were 3011 articles exported to Covidence, an online systematic review management platform. Through the hand-searching of Google Scholar and chaining other references, 37 more articles were found. A rapid review of titles was conducted in Covidence, with 2,131 titles were excluded because they did not specifically address social isolation or were not focused on COVID-19. Also, at the stage of reviewing abstracts, 892 abstracts were excluded because they did not focus on older adults, or concentrated on mixed populations, or were not focused on Africa. Therefore, the 25 titles had their full text downloaded, and 15 more were found ineligible because they were not empirical or were focused on other mental health issues, not SI/L. In conclusion (see Figure 1), 10 articles were deemed to be of high quality for review and exported to NVivo 12, a textual analysis software for content-based analysis.

### Data extraction

We adopted the JBI template for determining the quality of article, extracting details, characteristics, and results, together with the output of textual data coding on NVivo. This approach enhanced the robustness of the analysis and gave context to the presentation of data and discussions. With the JBI template, extracted ideas included—Author names, the title of the article, year of publication, country of publication, study Area, setting (rural or urban), research tradition, study design, research instrument, the unit of analysis, recruitment strategy, sample size, living arrangement of the study participants, age, gender, marital status, and education. Additionally, study objectives/research questions and research findings were curated. Finally, the JBI critical appraisal tool was also useful in assessing the quality (trustworthiness, relevance, and results) of the articles in an iterative process, from the rapid review of titles and abstracts to the final inclusion of the reviewed studies.

Furthermore, through the content analysis on NVivo 12, precisely 12 codes were generated through a deductive analysis process during the rapid review of the titles and abstract through discussions by AIA, AVW and JP, and five sub-codes were developed through an inductive process by AIA and AVW (see Table 1). We ensured the rigor of the data analysis process through researcher triangulation (32, 33) by comparing the perspectives of each researcher regarding the interpretation of the findings. Also, through a process of refutational analysis, we sought both convergence and divergence in the data (34), so that the report provided comparative insight into the experiences of SI/L in Africa. This approach provided insights into the context of SI/L among older adults in African communities.

**Table 1:** Textual coding of the articles.

**S/N**	**Main codes**	**Sub-codes**
1	Addressing social isolation	
2	The benefit of the social isolation-distancing	
3	Culture and Community	
4	Effect of isolation	
5	Family trauma-situation-challenges-dysfunction	
6	Future research	
7	Policy OR Program issues	
8	Reasons for social isolation	
9	Social isolation as a social problem	
10	Social support availability	
		Social support availability\Social Support—Community
		Social support availability\Social Support—Family
		Social support availability\Social support—Government
		Social support availability\Social support—neighbors
		Social support availability\Social support—Religion-Church
11	Social support is not available	
12	Technology use	

## Results

### Study characteristics

We first describe the characteristics of each article included in this review (see Table 2). Table 2 focuses on each article's basic characteristics, including the year of publication, country of research, and study setting. Also, we describe the research tradition, study design, sample size, and. In addition, we present the characteristics of the research participants (e.g., age, gender, education, occupation, and living arrangement, etc.,).

**Table 2:** Study characteristics.

**S/N**	**Authors**	**Title of article**	**Year of publication**	**Country of publication**	**Study area**	**Rural or urban setting**
1	Delali Adjoa Dovie	Pearls in the COVID-19 pandemic: The case of older adults' lived experiences in Ghana	2021	Ghana	Tema	Urban
2	Kemal Jemal, Tinsae Abeya Geleta, Berhanu Senbeta Deriba, and Mukemil Awol	Anxiety and depression symptoms in older adults during coronavirus disease 2019 pandemic: A community-based cross-sectional study	2021	Ethiopia	Debre Libanos District	Peri-urban
3	Thabang Manyaapelo, Anita Edwards, Nondumiso Mpanza, Samukelisiwe Nxumalo, Zama Nxumalo, Ntombizonke Gumede, Nothando Ngwenya, Janet Seeley	COVID-19 and older people's wellbeing in northern KwaZulu- Natal – the importance of relationships	2022	South Africa	uMkhanyakude District	Urban
4	Anthony Kwame Morgan, Justin Cobbold, Beatrice Aberinpoka Awafo, Daniel Katey, Theophilus Quartey and Rahinatu Ibrahim	COVID-19 and Psychological ‘Distress among Older Adults in Ghana	2021	Ghana	Accra and Kumasi Metropolitan areas	Urban
5	Prince Chiagozie Ekoh, Elizabeth Onyedikachi George and Chigozie Donatus Ezulike	Digital and Physical Social Exclusion of Older People in Rural Nigeria in the Time of COVID-19	2021	Nigeria	Ihe Community	Rural
6	Prince Chiagozie Ekoh, Patricia Ujunwa Agbawodikeizu, Chukwuemeka Ejimkararonye, Elizabeth Onyedikachi George, Chigozie Donatus Ezulike, and Ikechukwu Nnebe	COVID-19 in Rural Nigeria: Diminishing Social Support for Older People in Nigeria	2020	Nigeria	Ihe Community	Rural
7	Prince Chiagozie Ekoh, Elizabeth Onyedikachi George, Patricia Uju Agbawodikeizu, Chigozie Donatus Ezulike, Uzoma Odera Okoye and Ikechukwu Nnebe	“Further Distance and Silence among Kin”: Social Impact of COVID-19 on Older People in Rural Southeastern Nigeria	2022	Nigeria	Ihe and Umudioka Communities	Rural
8	Clarissa Giebel, Bwire Ivan and Isaac Ddumba	COVID-19 Public Health Restrictions and Older Adults' Wellbeing in Uganda: Psychological Impacts and Coping Mechanisms	2022	Uganda	Mukono District	Rural
9	Emmanuel Akwasi Asante, Kofi Awuviry-Newton, and Kwamina Abekah-Carter	Social Networks in Limbo. The Experiences of Older Adults During COVID-19 in Ghana	2021	Ghana	Jamestown Community	Urban
10	Prince Chiagozie Ekoh	Anxiety, isolation and diminishing resources: the impact of COVID-19 pandemic on residential care home facilities for older people in south-east Nigeria	2021	Nigeria	Anonymized carehomes in Enugu and Anabra States, Nigeria	Rural

Ten articles published between 2020 and 2022 were included in this review, and all articles focus on SI/L among older adults in the COVID-19 era. Six articles were published in Dovie (35), Jemal et al. (36), Morgan et al. (37), Ekoh et al. (22), Asante et al. (26), Ekoh (38). Also, three articles were published in Manyaapelo et al. (29), Ekoh et al. (28), Giebel et al. (14), and one was published in Ekoh et al. (39). Four of the included articles were focused on the social isolation experiences of older adults in Nigeria (22, 28, 38, 39), three were from Ghana (26, 35, 37), one each from South Africa (29), Ethiopia (36), and Uganda (14). The four Nigerian articles (22, 28, 38, 39) have the same first author, are results of different small studies conducted in the same communities (Ihe and Umudioka) and two anonymized care homes in Enugu and Anambra States, Nigeria. Furthermore, five studies were conducted in Urban and Peri-Urban settings (26, 29, 35–37).

#### Research design of included studies

As shown in Table 3, all of the studies utilized a cross-sectional design and there are two quantitative studies (36, 37) and the remaining eight articles were based on qualitative studies. Among the qualitative articles, three studies adopted the phenomenological method (26, 28, 35), four studies used in-depth interview guides (26, 28, 29, 39), while one study (38) involved a mix of older adults and caregivers as research participants, and the remaining studies focusing solely on older adults. The studies were all community-based, and two studies (14, 29) recruited participants and conducted the research through phone interviews.

**Table 3:** Research design of included studies.

**S/N**	**Research tradition**	**Research method**	**Research Instrument**	**Unit of analysis**	**Recruitment strategy**
1	Qualitative	Interpretive Phenomenology (Cross-sectional)	Individual interview guide	Older adults	Community-based, purposive
2	Quantitative	Cross-sectional	Standardized and pre-tested general anxiety disorder-7 and geriatric depression scale	Older adults	Community-based
3	Qualitative	Not stated	In depth interview guide	Older adults	Phone calls (Community-based)
4	Quantitative	Cross-sectional	Single-closed ended questionnaire	Older adults	Community-based
5	Qualitative	Qualitative design (Cross-sectional)	Semi-structured in-depth interview guide	Older adults	Community-based
6	Qualitative	Not Stated	In depth Interview guide	Older adults	Community-based
7	Qualitative	Phenomenological approach (Cross-sectional)	In depth Interview guide	Older adults	Community-based
8	Qualitative	Not Stated	Semi-structured interviews	Older adults	Phone call (Community-based)
9	Qualitative	Phenomenological approach (Cross-sectional)	In depth Interview guide	Older adults	Community-based
10	Qualitative	Not Stated	Interview guide	Older adults and female adult caregivers	Community-based

#### Characteristics of the research participants

Across all the included articles, information on Table 4 shows, there were 961 research participants, and the sample sizes ranged from 11 participants (22, 39) to 423 participants (36) across the studies. The minimum age of research participants was age 50 (37) and the maximum was 88 years (29). The majority (57%) of the participants are female and most (47%) older adults were not married. More than half (51%) of the older adults have acquired formal education and some (37%) older adults are retirees. Regarding living arrangements, research participants predominantly live within multigenerational households, spouses, or caregivers. However, three studies from Nigeria observed an emerging pattern of living alone or in residential care (28, 38, 39) and one from South Africa (29).

**Table 4:** Sample characteristics of research participants.

**S/N**	**Sample size**	**Living arrangement**	**Age**	**Gender**	**Marital status**	**Education**
1	10	Living alone, living with a caregiver and a daughter and her family, and living with spouses.	Range: 60 to 84 years	Males−5 Females−5	Married−4 Widow(er)−4 Divorced−2	Diploma−2 Middle school−4 First degree−3 PhD degree−1
2	423	Alone (46.5%) With family (53.5%)	60–70 (45.0%) 71–80 (30.6%) 81–90 (17.4%) >90 (7.0%)	Males−217 Females−206	Single−85 Married−159 Widowed−117 Divorced−62	No formal education−298 Has formal education−125
3	26	Most of the participants did not live with adult children.	Range: 61 and 88 years	Males−11 Females−15	Not reported	Not reported
4	400	Not reported	50–60 years (52.8%) 61–70 years (37.2%) Above 70 years (10%)	Males−151 Females−249	Married−229 Divorced−79 Widowed−92	No formal education−70 Basic education−124 High school−99 Tertiary education−107
5	11	4—Lived alone, 7—Lived with family, spouse and/or children).	60–70 (5) 71–80 (5) 81–90 (1)	Males−5; Females−6	Not reported	No formal education−5 Primary education−4 Secondary education−2 Undergraduate education−1
6	11	2—Lives with their spouses and children 1—Lives with children 8—Live alone	Range 60–81	Males−4; Females−7	Not reported	No formal education−4 Primary education−4 Secondary education−2 University education−1
7	20	13—Live alone; 5—Lives with spouses; 2—Lives with spouses and children	Not reported	Males−8; Females−12	Not reported	No formal education−7 Primary education−9 Secondary education−3 University education−1
8	30	Household size−2 to 8 family members−23 (Resided with family members—grandchildren)	Not Reported	Males−7; Females−23	Not Reported	Not reported
9	15	9—Living with spouse and children 6—Live alone	60−70 (9) 71−80 (6)	Males−5 Females−10	Married−9; Widowed−5; Divorced−1	No formal education−13; Elementary school−2
10	15	Residential care home	66−78 for older adults; 39−58 Caregivers	Males−3; Females−7; Caregivers (Females−5)	Not reported	

### Social isolation facilitators among older adults in Africa

Several articles identified immediate and indirect reasons for the experience of social isolation among older African adults. The primary cause of social isolation among older African adults were the restrictive COVID-19 public health guidelines to protect older adults, travel restrictions, a ban on gatherings, and physical/social distance. Older adults were at high risk of infection and death due to COVID-19, such that family and social networks of support were careful not to visit (36, 39). Thus, the need to keep a social distance was a significant reason for social isolation in the research reviewed (14, 29, 35, 36, 39). In addition, the restriction on vehicular movement across locations, primarily between urban and rural destinations (22, 29) made it difficult for potential informal sources of social support to return home and connect physically with older adults. This disconnect was more acute among older adults who lived alone (28). Also, the ban on gatherings (22, 26, 28, 29) impeded the social connections that older adults would have with the large community.

An indirect cause of social isolation was the economic downturn, which caused financial pressures due to job/livelihood loss and unaffordable transportation costs (22, 28, 29). In addition, the economic downturn made it difficult for adult children and other sources of support to visit their parents and send them gifts, being part of the cultural expectations from adult children in African settings.

### Loneliness facilitators among older adults in Africa

The environmental and emotional triggers of loneliness among older adults primarily resulted from the social isolation they experienced. Similar to social isolation, the environmental triggers of loneliness among older adults in Africa included the metaphor of “staying away to protect older adults from the high risk of infection and mortality” (22, 26). Also, during economic downturn, the ban on vehicular movement, and sociocultural activities caused job/livelihood loss and a high cost of transportation, which contributed to feelings of disconnection. These factors also led to the inability of relevant social networks to organize socials, visit, send gifts, and provide money, creating a feeling of loneliness (29). In addition, a peculiar feeling of loneliness occurred among older adults who lived within a family structure. This form of loneliness was observed among participants who had a family trauma, such as the death of a child or spouse or divorce/separation (26, 29). The feeling of loneliness was mostly expressed by male older adults who do not share common social activity interests with members of their household (26). Loneliness occurred when children and wives had an interest in television or phones when the male older adult enjoyed a game like “Oware (a popular game in Ghana)” with friends and associates.

### Effects of social isolation and loneliness among older adults in Africa

Social isolation and loneliness due to COVID-19 in Africa affected older adults' mental, communal, spiritual, financial, and physical health. Social isolation and loneliness among older adults in Africa were associated with depression among older adults (36). Consequently, lonely older adults reported a decline in cognitive capacities because their minds were not socially stimulated, potentially increasing the risk of cognitive impairments like dementia (14). Also, the experience of social isolation and loneliness tested Africa's cultural philosophy of interconnectedness, togetherness, and relationships within communities (29). In addition, older adults' spirituality was affected by social isolation due to a lack of congregation, especially if they were religious adherents before the lockdown (35). The economic effect of social isolation was that some older adults could not visit their plots of land to garden and grow vegetables as a means of subsistence (14). In similar situations, older adults could not access support with household chores and farming activities (39). As a result of the disconnection from financial activities, there was also a decline in physical activities. Besides, older adults with multiple morbidities and physical disabilities were at risk of health complications and death (38). Furthermore, those who were frail before COVID-19 became frailer because they could not perform regular physical activities (14).

### Coping with social isolation and loneliness among older adults in Africa

Several articles documented ways older adults coped with social isolation and loneliness associated with the COVID-19 restrictions. The use of technology was vital, as was the role of social networks within the family, community, religious groups, and government.

#### Technology adoption

Technology helped some older adults in Africa to cope with isolation, such as platforms including WhatsApp and telephone calls (35), and Facebook and Twitter for social contact and information (29). Also, television and radio help stay in touch with the outside world through news and other programs (22). In addition, older adults played video videogames on the Internet and engaged in video calls to overcome boredom (35). Conversely, regardless of these benefits, many barriers were identified in the review. For example, some older African adults did not feel that the benefits of technology were adequate, noting that technology could not replace the value of face-to-face interactions (26). Significantly, the digital divide problem affected older adults due to the lack of access to the Internet, digital skills, and negative disposition toward technology. Other problem areas were the cost of devices, the quality of the infrastructure, and personal opinions about technology (22).

Moreover, many older adults in Africa did not consider using technology as effective in reducing their loneliness. Instead, they cited reasons such as being used to living without phones and that phones do not provide any social engagement (22). In another case, older adults expressed concerns about the lack of infrastructure for smartphones, including electricity and Internet access (14). For instance, the weak infrastructure in rural areas affected the quality or duration of telephone communication (22, 29).

### Available social support

Research also indicates that many older adults could cope with the social isolation and loneliness linked to the COVID-19 pandemic by leveraging social connections through family members, religious groups, and the government to support their physical and emotional needs.

#### Familial and community support

For older adults who lived within family structures and communities, schoolchildren were available at home because schools were shut down, and they provided social support and engagement to older adults (35). Also, adult children were available to provide support, although some had lost their livelihoods. Regardless of the economic burden of supporting adult children's daily needs, Manyaapelo and colleagues emphasized that older adults valued the sense of family and community. This reduced the potential stress and pressure of the COVID-19 period (29). In cases where the older adult lived alone, adult children who sent financial or material support or gifts provided older adults with a sense of social connection (29). In other cases, some older adults relied on the support of their extended family members, such as uncles, nieces, and nephews, to help them maintain a livelihood. The extended family members provided the support because that was the role expected of them in the local culture (29).

On the other hand, the family as social support did not work well for some older adults who experienced family trauma and conflict. Familial support was not forthcoming when there was a sick adult child, divorce/separation, death of an adult child, or adult children abandoning their parents (29).

#### Role of religious groups

Several articles also identified religious groups as a source of support by visiting older adults in the communities, and making food available to people living in the community (22, 29). After the initial effect of the lockdown, religious groups developed innovative ways to address the need for staying connected with members. They also set up smaller groups to meet up with older adults in their homes and at the community level. Thus, even when the gathering in the church was impossible, older adults living within the communities were reached by religious organizations (28).

#### Government support

The support from the government varied considerably across countries. In the included articles, the provision from governments in South Africa and Nigeria were identified as more supportive. In South Africa, apart from the Pension scheme, the government provided a Special COVID-19 Social Relief of Distress to people considered qualified to access this support (29). South Africa has a well-structured system of providing social support to older citizens and this was utilized during the outbreak. Similarly in Nigeria, there exists a pension scheme with limited coverage for older adults. In addition, the government set up a COVID-19 Palliative Care System through the direct distribution of food. However, there is evidence that the food was not equally distributed (39), mainly because rural areas are usually disproportionately excluded from social services and support. In Ghana, the Social Security and National Insurance Trust was not functioning appropriately, hence there were prolonged delays in payment. This situation was aggravated during COVID-19 and older adults could barely get one payment in 5 months (26).

### Research gaps

The 10 studies in this review reveal methodological challenges such as the risk of selective survival bias, sampling biases, and limited inductive value due to context. The risk of selection bias in the study occurred because the sampling was conducted online, and there was a tendency to exclude older adults who were not literate or could not use the computer (37). Also, the potential for sampling bias exists, primarily due to the limited number of study sites (36) and the exclusion of older adults without mobile phones (29).

A significant research gap in African social isolation and loneliness studies is the lack of large-scale quantitative and longitudinal studies (14) to capture the experiences of older adults during COVID-19. Topical research interests include the unmet social and functional needs (39), mental health, the digital divide (14, 28, 35), the effect of social networks (26, 35), and the rural-urban dichotomy of SI/L (14). In particular, there is a need to support research to explore diversity across all African countries, and this could be fostered through the development of comprehensive, collaborative research networks. There is also a need for evaluation research to assess the efficacy and effectiveness of various programs and services to improve social connectedness among older African adults living in diverse settings.

### Policy gaps

The reviewed articles highlighted important policy gaps for African mental health support services, media programs, and community care service integration targeting older adults. It was clear that mental health care is floundering in Africa. The psychological distress among older adults at the height of the pandemic and the inability to access help constituted high risk and lower adaptation (37). Some research suggests the need to have mental health hotlines that will provide virtual menta health services (22). Also, regarding media programing, older adults in the reviewed studies interacted with TV and radio as means of staying connected when they are unable or unwilling to use digital devices. Radio and TV programming should present older adults in Africa with age-friendly programs that will keep them engaged (22, 28). Additionally, because the pandemic has tested the capacity of Africa's informal support structures, the government must integrate caregivers and social workers into a care network at the community level (28, 39). Another approach to integrating services at the community level is a partnership and providing grants to residential care facilities as an emergent support structure.

## Discussion

COVID-19 restrictions were similar across countries, and in Africa, the experience of SI/L was acute because of the disconnect of kinship-based care and support (3, 4) and the lack of African governments' commitment to providing social support to older adults (1). Countries with more developed welfare programs did not rely as heavily on kinship networks. Therefore, regardless of the benefits of African social organization based on a common bond and communalism, these were unsustainable during the COVID-19 pandemic lockdowns. Comparatively, because welfare states have protective characteristics that address SI/L (40, 41), they fared better than the African countries lacking system-level strengths.

Thus, a challenge of the African kinship-based support structure is that it triggers a feeling of isolation when disconnected. Isolation occurs because the kinship approach creates a sense of expectation (41) due to the norm of reciprocity between parents and children (42). Therefore, the experience during COVID-19 confirmed the assertion that older adults in individualistic social settings experience a lesser sense of loneliness than in collectivist societies (43). Yet, regardless of society type, the physical and mental health effects of SI/L (12) include frailty, early dementia onset (14), psychiatric conditions (e.g., depression and anxiety), and severe cognitive impairments (13). Similarly, loneliness can exacerbate physical conditions like obesity and cardiovascular diseases (16, 19).

During the pandemic, technology adoption was a key tool in addressing SI/L in many countries globally (24, 44). In African literature, the smartphone was the common technology, followed by television and radio. However, the telephone was more beneficial because it assisted older adults in speaking with family members through video and voice calls (22), considering that such technologies had been in use among older adults before the pandemic. Unfortunately, there is currently no data in Africa that compares the rate of technology adoption among older adults before and during the COVID-19 pandemic. However, it is easy to infer that the rate of technology was low due to contextual considerations. For example, older adults were unwilling to acquire digital skills, experienced digital infrastructure barriers, and thought smartphones did not provide as many social connections as they desired (22, 26, 45). Comparatively, in the era of COVID-19, the technology adoption rate increased in a place like Canada, albeit similar problems occurred but at lower rates [e.g., (24, 46)]. So, there was a consensus in the research about the lack (though in various degrees) of digital infrastructure, cost of devices, and Internet coverage in African and western countries. These claims are valid, and the barriers to technology specifically suited to the needs of older adults must be addressed to promote higher social connectedness in future outbreaks. The challenges of digital skills and infrastructure can be addressed by engaging the private sector and encouraging the development of frugal innovation because frugal innovation promotes less engineering complexity and low cost of production and maintenance. So, frugal innovation will ensure that the technologies for Africa are designed and developed based on the infrastructural realities and interests of Africa and Africans.

In sum, the available social support during the pandemic was family, community, religious groups, and government support. These same support areas have been documented in studies conducted outside Africa (5, 47). Regardless of the entrenched communalism and common-bond ideologies in Africa, non-African literature captured how support was made available to older adults during the pandemic. The role of government was effective outside of Africa because those countries had well-developed welfare regimes that catered to the needs of older adults (40). Neither the Pension scheme nor COVID-19 palliative measures were effective in most African countries because the database infrastructure was inefficient. An additional problem was the historical rural-urban divide regarding the imbalances in the spread of infrastructure (14, 22, 29). By implication, the systemic weaknesses further complicated the experiences of SI/L among older adults in Africa. Since familial support was severely hit in a similar measure across all the countries, the ability of the government to provide practical medical, social, and material support varied greatly between places. Therefore, given the lack of comparative evidence, it is safe to assume that older adults in other parts of the world fared better during the pandemic because government support was available. Future researchers should consider geographically comparative research, which is needed to answer these questions.

There were important research and policy gaps regarding the experiences of COVID-19 among older adults in the African literature. Regarding the research gap, there have been relatively few large-scale research projects (mixed methods, qualitative, and quantitative) in Africa documenting older adults' social, physical, and mental health experiences through the COVID-19 pandemic. Hence, the evidence is sparse. The World Health Organization's Regional Office for Africa report also recognizes these gaps. In addition, a large proportion of evidence emerged from a few select African countries, including Ethiopia, Ghana, Nigeria, South Africa, and Uganda. Unfortunately, these are just too few to drive the evidence from Africa and allow researchers to make claims about SI/L experiences broadly. Hence, the WHO's 2021 report on the impact of COVID-19 on older African adults relied on case studies rather than community experiences (1). Therefore, it is essential to conduct empirical research to explore the challenges older adults experienced during the COVID-19 restrictions. Also, such data will help identify the changes over time, especially because the pandemic has historical value. In addition, the outcome of such research will provide the basis for policy intervention in the core areas of unmet needs and assistance to resource management planning for governments in the future.

The COVID-19 pandemic also exposed weaknesses in the policy framework for caring for older adults in many Africa countries. For example, in Nigeria, during the pandemic, the government's inability to adequately support the vulnerable population, including older adults, triggered the restart of the National Identification registration system, which had been redundant since 1999. In addition, to addressing the national registration issues, it was essential to consider integrated healthcare services at the community level. Community-level support for mental, social, and physical wellbeing support was absent in most African countries. An integrated approach is necessary because the communal approach to care was unsustainable during the height of the pandemic. Therefore, it is vital to consider complementing the existing care structure through policies and programs that operate at the community level. An example of successful community-integrated efforts during COVID-19 was in Vietnam (48), where grassroots health networks consisting of district hospitals, health centers, health stations, and village health collaborators led the community-level engagements with the people. Such models will be valuable for building strong and resilient support systems for older African adults, given the network-oriented nature of African communities.

## Limitations

The African focus of this scoping review is restricted because only a few studies were available from Africa, as there were only five African countries (Ethiopia, Ghana, Nigeria, South Africa, and Uganda) involved in the review. Only a limited number of studies describe the COVID-19 experiences of older adults in Africa. Seven articles reported studies conducted in Nigeria (4 articles) and Ghana (3 articles). Thus, the review can only focus on West Africa. Also, most of the included articles were based on qualitative studies. Therefore, it was impossible to derive population-wide estimates of essential associations in quantitative terms. Also, the rural-urban dichotomy of the study areas is subject to debate globally because there is no consensus about the definition of rurality or urbanity. Finally, due to the paucity of large-scale examples, this review was limited in comparing Africa with other continents to provide insight into the causes and consequences of SI/L among older adults during COVID-19 (31).

## Conclusion

This is the first known review exploring the social isolation and loneliness of older adults in Africa during the COVID-19 pandemic. Similar to many countries which enacted COVID-19 lockdown policies, the experience of SI/L among older adults in Africa was primarily caused by the lockdown restrictions. In African countries, these lockdown policies resulted in a severance of older adults from the cultural structure of care for older adults and their familial support systems. Weak government intervention, the existing rural-urban disproportionate spread of infrastructure and social welfare services, personal situations, challenges regarding technology, and detachment from daily activities, disproportionately affected older adults in Africa. Care structures like the family and community could not implement the required intervention. SI/L among older adults in Africa had mental and physical health challenges outcomes which integrated care service at the community and government support could have addressed if they were available. It is important to address the research and policy gaps to capture the spectrum of COVID-19 experiences and provide intervention for older adults.

## Data availability statement

The raw data supporting the conclusions of this article will be made available by the authors, without undue reservation.

## Author contributions

IA: main writer of manuscript and review of articles. AW: reviewed and edited manuscript. JP: secondary review of articles and editorial support. All authors contributed to the article and approved the submitted version.
