# Supplementary material for: COVID-19 experiences of social isolation and loneliness among older adults in Africa: a scoping review
Source: Front Public Health. 2023 May 9;11:1158716. doi: 10.3389/fpubh.2023.1158716 (PMC10203559; doi:10.3389/fpubh.2023.1158716)
Supplement: Supplementary file 1 [file Table_1.pdf]

Appendix 1: Search Strategy

S/N	Database	Search Term	Search Period	Results
1	CINAHL Complete	Africa AND (older AND adults OR aged OR elderly OR seniors) AND (social AND isolation OR loneliness) AND (covid-19 OR coronavirus OR 2019-ncov OR sars-cov-2 OR cov-19)	2020 - 2022	795
2	APA PsycINFO	Africa AND (older AND adults OR aged OR elderly OR seniors) AND (social AND isolation OR loneliness) AND (covid-19 OR coronavirus OR 2019-ncov OR sars-cov-2 OR cov-19)	2020 - 2022	721
3	SCOPUS	("africa" AND older AND adults OR aged OR elderly OR seniors AND social AND isolation OR loneliness AND covid-19 OR coronavirus OR 2019-ncov OR sars-cov-2 OR cov-19)	2020 – 2022	993
4	Web of Science	(ALL=(("africa" AND older AND adults OR aged OR elderly OR seniors AND social AND isolation OR loneliness AND covid-19 OR coronavirus OR 2019-ncov OR sars-cov-2 OR cov-19))) AND (PY==("2020" OR "2021" OR "2022"))	2020 - 2022	2095
5	Ageline	"Africa" AND older AND adults OR aged OR elderly OR seniors AND social AND isolation OR loneliness AND covid-19 OR coronavirus OR 2019-ncov OR sars-cov-2 OR cov-19	2020-2022	162
6	PubMed	((("Africa") AND (older AND adults OR aged OR elderly OR seniors)) AND (social AND isolation OR loneliness)) AND (covid-19 OR coronavirus OR 2019-ncov OR sars-cov-2 OR cov-19)	2020-2022	253
